# Topics of the Month

**Published:** 1896-07

**Authors:** 


### TOPICS OF THE MONTH.

At the French Congress of Ophthalmology, recently held at Paris, Dr. Lucien Howe, of Buffalo, presided during its final meeting, May 7, 1896. It has been said that French physicians are jealous of their foreign confrères, but the choice of Dr. Howe to occupy the chair at the last session of this congress must be regarded not only in the light of a denial of such charge, but as a mark of courtesy to the United States. Dr. Howe, accepting the office in that spirit, declared that he looked upon it rather as an honor to his nation than to himself. He brought forward the question of precautions to be taken to prevent ophthalmia of the new-born, citing the fact that in several of the American states legislative action has been taken looking to the punishment of midwives if they do not give prompt notice to the public health authorities whenever this disease occurs within their knowledge. The French do not seem to think it possible to pass such laws in France, though they admit that the punishment of a few offenders would prove of benefit.

---

In another column we publish a notice of an examination to fill vacancies in the medical corps of the United States army. These vacancies offer a splendid opportunity for young men and they should be filled readily. In the present state of medical education recent graduates leave school better equipped than formerly, and they ought to find themselves well prepared for the army, navy, and marine hospital service examinations.

